# Transcription factor gene *Pea3* regulates erectile function during copulation in mice

**DOI:** 10.1371/journal.pone.0276069

**Published:** 2022-10-27

**Authors:** Jarret A. P. Weinrich, Aanchal Tyagi, Megan C. Kenney, Richard J. DiCasoli, Julia A. Kaltschmidt

**Affiliations:** 1 Biochemistry, Cell and Molecular Biology Program, Weill Cornell Graduate School of Medical Sciences, Weill Cornell Medical College of Cornell University, New York, New York, United States of America; 2 Developmental Biology Program, Sloan Kettering Institute, New York, New York, United States of America; 3 Wu Tsai Neurosciences Institute, Stanford University, Stanford, California, United States of America; 4 Department of Neurosurgery, Stanford University School of Medicine, Stanford, California, United States of America; UiT Norges arktiske universitet, NORWAY

## Abstract

Male mice with homozygous loss of function mutations of the transcription factor gene *Pea3* (*Pea3* null) are infertile due to their inability to inseminate females, however the specific deficits in male sexual behaviors that drive this phenotype are unknown. Here, the copulatory behavior of male mice (*Pea3* null and control) with hormonally primed ovariectomized females was monitored via high-speed and high-resolution digital videography to assess for differences in female-directed social behaviors, gross sexual behaviors (mounting, thrusting), and erectile and ejaculatory function. *Pea3* null male mice exhibit greatly reduced erectile function, with 44% of males displaying no visible erections during copulation, and 0% achieving sustained erections. As such, *Pea3* null males are incapable of intromission and copulatory plug deposition, despite displaying largely normal female-directed social behaviors, mounting behaviors, and ejaculatory grasping behavior. Additionally, the organization and timing of thrusting behaviors is impaired in *Pea3* null males. Our results show that the transcription factor gene *Pea3* regulates the ability to achieve and maintain erections during copulation in mice.

## Introduction

For males, successful copulation involves the tight coordination of context-dependent social behaviors, erectile functioning, and ejaculation. Approximately 20–50% of men may suffer sexual dysfunction [1, 2], which can range from diminished sexual arousal and erectile function to the complete absence of erections and ejaculations [3]. First line treatments for these patients often fail [4], therefore there is a need to advance the current understanding of the molecular pathophysiology of erectile dysfunction to aid in the development of novel therapeutics. Given that transcription factors are primary regulators of the complex genetic and developmental pathways that support normal organ function, there has been recent emphasis on discovering linkages between transcription factors and human diseases [5, 6]. To date, only a handful of transcription factors have been identified that are linked to male sexual dysfunction [6–8]. However, it is unknown whether these genes directly participate in organizing erectile or ejaculatory function during active copulation or if they mainly alter sexual behavior in other ways (i.e., by diminishing sexual arousal [9]). Therefore, there is still not a clear delineation as to how transcription factors regulate specific facets of male sexual behavior.

Male mice with a homozygous loss of function mutation of the *ETS* (*E twenty-six*) class transcription factor gene *Pea3* (*Pea3* null) are completely infertile, which is thought to be due to ejaculatory dysfunction, as *Pea3* null mice fail to deposit detectable amounts of sperm within the female reproductive system or copulatory plugs [8]. In adult mice, *Pea3* is expressed within male reproductive organs (testis and epididymis), suggesting a possible role for *Pea3* in spermatogenesis or maturation [8, 10]. However, *Pea3* null males produce sperm capable of fertilization as determined by *in vitro* assays [8]. Similarly, *Pea3* null mice do not exhibit any identifiable histological deficits in penile tissue, have intact corpus cavernosum contractile ability during the application of excitatory neurotransmitters in *ex vivo* assays, and are capable of producing reflex erections, suggesting that erectile functioning is intact [8]. Furthermore, during the initial characterization of the *Pea3* null sexual behavior phenotype, Laing *et al*. witnessed *Pea3* null males performing mounting behaviors at the start of overnight mating assays, suggesting that both appetitive and consummatory male sexual behaviors are at least partially intact [8]. A systematic characterization of male sexual behaviors, including direct observation of the penis during copulation, of *Pea3* null male mice has yet to be performed. As such, the specific behavioral deficits that accompany, and potentially drive, the abolishment of insemination and copulatory plug deposition in these mice are unknown.

Here, we hypothesized that the *Pea3* null male infertility is due to 1) severe erectile dysfunction during copulation and/or 2) the complete loss of ejaculatory function. To ascertain deficiencies in *Pea3* null male-specific female-directed social behaviors, gross sexual behaviors, and erectile and ejaculatory functioning, we employed a sexual behavior arena [11] that allowed for direct observation of erectile function and monitored mouse behaviors using high speed and high resolution digital video recording, which was followed by a detailed post hoc analysis. We found that loss of *Pea3* leads to greatly diminished erectile functioning, with many mice displaying no visible erectile activity, and those that do only produce brief, rare, and poorly timed periods of visible erectile activity. Additionally, we found that the coordination of hip thrusting patterns during sexual behavior is perturbed in *Pea3* null males. These results indicate that the transcription factor *Pea3* is an important regulator of erectile functioning during copulation in mice.

## Materials and methods

### Animal husbandry

All mouse husbandry and surgical procedures strictly adhered to the regulatory standards of the Institutional Animal Care and Use Committee of Memorial Sloan Kettering Cancer Center (MSKCC; protocol 08–06-009). The following mouse strains were used in this study: *Pea3^NLZ^* mice (also known as *Etv4^tm1Arbr^* [12]) and ovariectomized c57BL6/J females (Stock: 00064; Jackson Laboratories; 41 females total). Control male mice were wildtype littermates of the *Pea3* null mice. The health and wellbeing of the mice was monitored daily by trained staff of the MSKCC animal facility. At the completion of the study, mice were euthanized by carbon dioxide inhalation or processed for further use by intracardial perfusion with 4% paraformaldehyde under ketamine/xylazine anesthesia.

### Preparing for behavioral testing

Sexual behavior was conducted as by Park [11] and Juntti *et al*. [13]. Briefly, male sexual behavior was assessed in a closed, rectangular plexiglass arena with a clear plexiglass bottom, below which an angled mirror was placed to allow for viewing of the mouse underside during the behavioral assay [11]. Both male and female mice were prepared for behavioral testing days in advance. Males were singly housed for at least one week before testing, and contact with cages (i.e., cleaning, etc.) were minimized. On each of the two days before testing, ovariectomized females were hormonally primed with a subcutaneous injection of 1 microgram of estradiol benzoate in sesame oil. 4 to 6 hours before testing, ovariectomized females were hormonally primed with a subcutaneous injection of 100 micrograms of progesterone in sesame oil. Ovariectomized females were used for multiple rounds of testing, and allowed to rest for at least one week after hormonal priming and/or behavioral testing [14, 15]. One hour before recording, males were transferred to the plexiglass arena and allowed to acclimate to the testing environment. Testing was only performed during the dark phase of the light/dark cycle set by MSKCC animal facility staff.

### Behavioral testing

Following acclimation to the testing arena, digital recording was initiated, and the hormonally primed female was introduced to the testing arena. Behavior was recorded with a Photonfocus 2048X1088C Series camera with NORPIX Streampix software, at a frame capture rate of 60 frames per second. If males did not commence sexual behavior within 15 minutes of introducing the female, the female was exchanged for an untested female until the male was introduced to 3 females. Behavior was recorded until the male ejaculated or within one hour of introducing the last female to the cage, whichever occurred first. Following the termination of testing, the females were checked for semen plugs and then placed into a separate cage from non-tested females. Males were placed back into their home cage. The behavioral arena was carefully washed with soap and water and dried before the next use.

### Video data quantification

Behavioral videos were manually scored within NORPIX Streampix software, with the scorers blind to the genotype of the male in each recording. Relevant data were recorded in excel and when possible the frame number for the start and end of the specific behaviors analyzed were included. Sexual behaviors were classified as follows: (a) mounting: the male positions himself parallel to the female’s body axis and grasps the female’s flank with his forearms (Fig 2H), (b) thrusting: repetitive oscillations of the male’s hips while in the mounting position (thrusting behaviors such as probing, intromission, and pauses are detailed below), (c) intromission: insertion of the penis into the vagina, and (d) ejaculatory grasping: during ejaculation, the male tightly grasps the female and falls over onto one side (Fig 2I). The following data were recorded: introduction of the female to the behavioral arena (time), start of each mount (time), the presence or absence of visible erections, the number of intromissions per mount, and the start and end of ejaculatory grasping (time).

Thrust duration and timing were analyzed in more detail for the last mount of each sequence, where a single thrust was defined as the beginning of one thrust to the beginning of the next thrust. Using similar definitions as those employed by Elmore & Sachs [16], thrusting behaviors were further defined by the time duration of the thrust, including: 1) probes, which are fast thrusting behaviors where the penis is seeking the vaginal opening (<0.2s); 2) intromissions, where the penis is inserted into the vagina (0.2–0.9s); and 3) pauses, which are longer duration periods where the male remains in the mounting position in between probing and intromission thrusts (>0.9s). For *Pea3* null mice we use the term “intromission-like” to describe various components of intromission associated behaviors (i.e., count, timing, etc.), as, by definition, intromission implies the insertion of the penis into the vagina. To generate the transitional probability matrix, first order transitional probabilities [17] were generated for each mouse, and averaged across mice for each genotype.

Social behaviors were assessed as in Fairless *et al*. [18]. Briefly, the interaction of the male with the female was assessed by measuring the cumulative time the male spent sniffing the nose, body, or anogenital regions of the female. The time the male spent grooming the female was also measured. The start and end time of each behavior was recorded to determine the cumulative time for each behavior. Behaviors were measured until the first mount occurred or for the first 15 minutes after the placement of the female in the behavior arena, whichever occurred first.

### Data analysis and statistics

Data were processed and analyzed in Mathwork’s MATLAB (R2020a) software. All indicated statistical tests were performed with Prism (GraphPad) statistical analysis software. The threshold for significance for all statistical tests were set at p < 0.05, and indicators of significance levels were as follows: ns (no significance) when p > 0.05; (*) when p < 0.05; (**) when p < 0.01; (***) when p < 0.001; and (****) when p < 0.0001. Data in figure legends are reported as the mean value ± the standard error of the mean.

## Results

### Female-directed social behaviors unaffected in *Pea3* null mice

**Fig 1:**
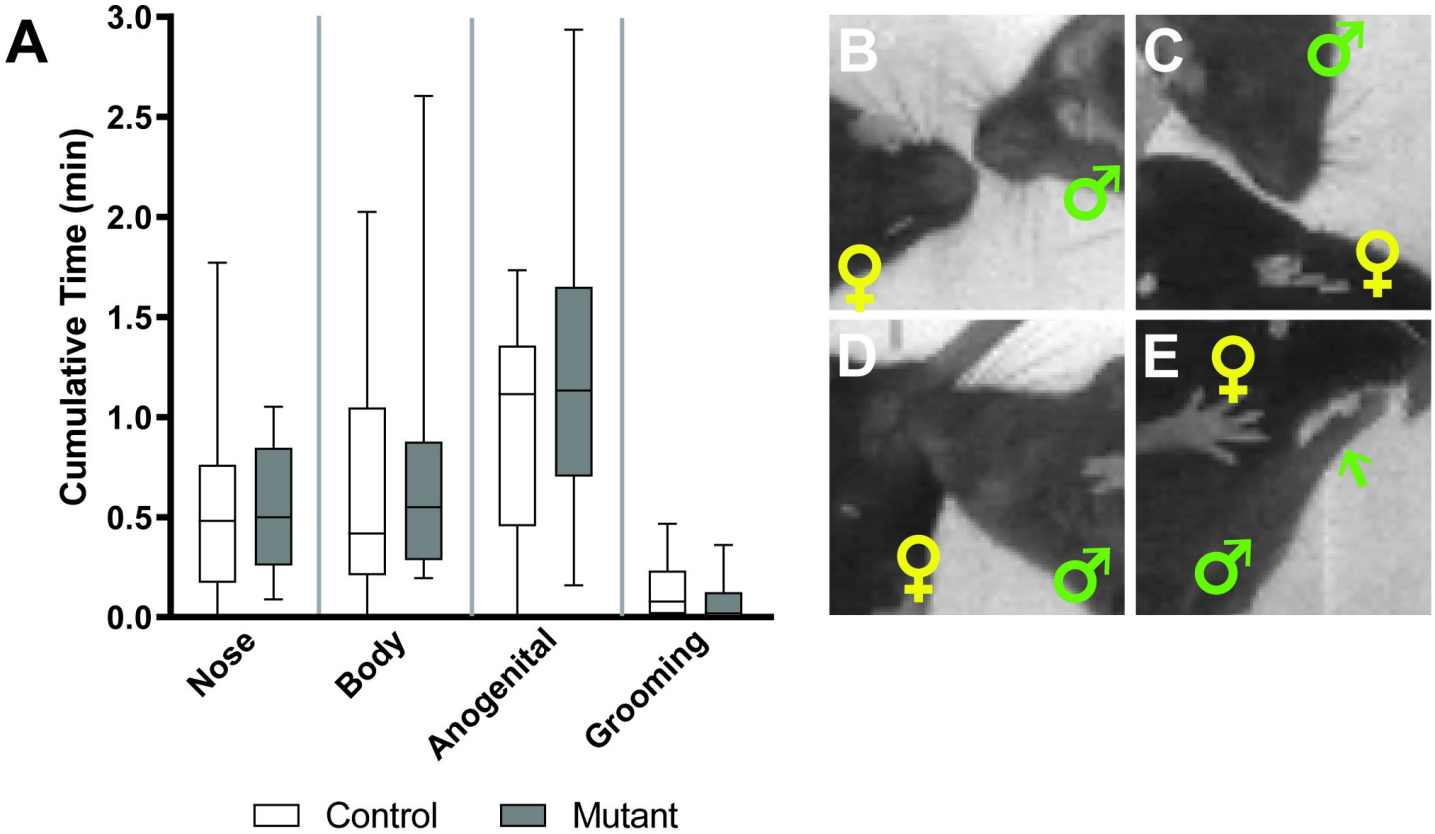
Female-directed social behaviors are unaffected in *Pea3* null mice. (A) The cumulative time spent performing social behaviors in unaffected in *Pea3* null (N = 17) vs control (N = 13) males (multiple Mann-Whitney tests, p_nose_ < 0.483; p_body_ < 0.408; p_anogenital_ < 0.432; p_grooming_ < 0.146). Representative images of active social behaviors directed to the female nose (B), body (C), and anogenital (D), and grooming (E) behaviors. Male and female positions are marked by green and yellow symbols, respectively. In (E), the green arrow denotes position of male forelimb during grooming.

First, we set out to determine whether the inability of *Pea3* null mice to set semen plugs was due to a decreased or abolished drive to interact with receptive females, thereby reducing non-sexual aspects of mating behavior. To this end, the duration of time males spent sniffing various regions of the female, specifically the nose, body, and anogenital region, and additionally, the duration of time of female-directed grooming, was quantified [18] (Fig 1B–1E). There is no difference in the cumulative time spent performing social behaviors between control and *Pea3* null mice (Fig 1A). Therefore, decreased female-directed behaviors do not underlie the loss of the ability of *Pea3* null males to deposit semen plugs.

### Gross sexual behaviors largely intact in *Pea3* null mice

**Fig 2:**
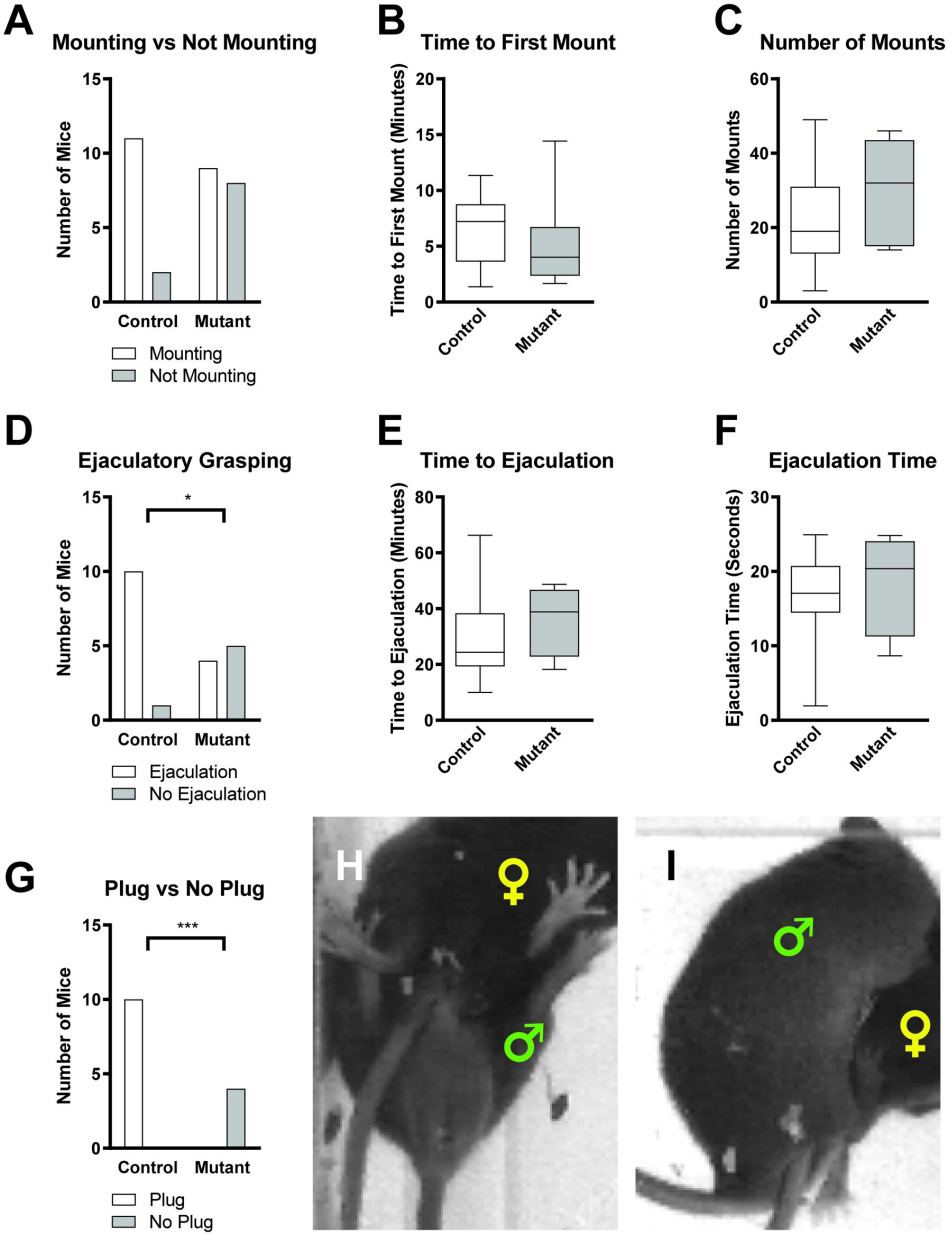
*Pea3* null mice exhibit deficiencies in ejaculatory behaviors. (A) The number of mice that perform mounting behaviors is not statistically significant between control and *Pea3* null mice (control: N = 13 mice; null: N = 17 mice; Fisher’s exact test, p < 0.1194). (B and C) There is no difference between control and *Pea3* null mice in time to first mount (B: control: 6.32±0.93 minutes, N = 11; null: 5.05±1.37 minutes, N = 9; Mann-Whitney test, p < 0.2947) and the number of mounts performed (C: control: 21.45±4.25 mounts, n = 11; null: 29.44±4.49 mounts, N = 9; Student’s t-test, p < 0.2142). (D) Fewer *Pea3* null mice perform the ejaculatory grasping behavior compared to control mice (control: N = 11 mice; null: N = 9 mice; Fisher’s exact test, p < 0.0498, *). (E and F) There is no difference between control and *Pea3* null mice in time to ejaculatory grasping (E: control: 30.01±5.04 minutes, N = 10 mice; null: 36.16±6.47 minutes, N = 4 mice; Mann-Whitney test, p < 0.5395) and the duration of ejaculatory grasping (F: control: 16.41±1.97 seconds, N = 10 mice; null: 18.58±3.51 seconds, N = 4 mice; Mann-Whitney test, p < 0.4535). (G) Of mice that perform ejaculatory grasping behavior, no *Pea3* null mice deposit copulatory plugs compared to all control mice depositing copulatory plugs (control: N = 10 mice; null: N = 4 mice; Fisher’s exact test, p < 0.001, ***). Representative images of mounting (H) and ejaculatory behavior (I). Male and female positions are marked by green and yellow symbols, respectively.

Next, a detailed analysis of gross sexual behaviors was performed to ascertain whether the inability of *Pea3* null mice to plug was due to a decreased propensity to mount receptive females. While there were fewer *Pea3* null males that mounted females, this difference was not statistically significant (Fig 2A). Of both control and *Pea3* null mice that did mount, we found that there was neither a difference in the time it took mice to perform their first mount (Fig 2B) nor in the number of mounts performed (Fig 2C). This indicates that the gross sexual motor behaviors are largely intact in *Pea3* null male mice.

One explanation for the loss of the ability to deposit semen plugs is that ejaculatory behavior was abolished in *Pea3* null males [19, 20]. We assessed whether mutant mice performed the stereotypical ejaculatory grasping behavior, during which the male stabilizes the female during insemination by grasping the female and falling over to one side (Fig 2I). We found that *Pea3* null males were still capable of performing ejaculatory grasping behaviors, albeit at a much lower frequency than control males, thereby ruling out delayed or absent ejaculation-related behaviors (Fig 2D). Additionally, *Pea3* null mice that perform this ejaculatory grasping did so within the same amount of time and for the same duration as control males (Fig 2E and 2F), indicating the ejaculatory grasping behavior was of a sufficient time span necessary to deposit a semen plug.

Despite performing mounts and ejaculatory grasping behavior, we confirmed that *Pea3* null males fail to deposit copulatory plugs within the vagina (Fig 2G), as previously reported [8]. Additionally, we did not witness expulsion of semen or copulatory plug from the penis of *Pea3* null mice at any time during our mating assays. Therefore, the lack of plug deposition was not due to aberrant extra-vaginal placement [21].

### *Pea3* null mice exhibit greatly diminished erectule function during copulation

Since *Pea3* null males display sufficiently normal gross mating behavior, yet fail to inseminate females, we next focused our analysis on the placement of the penis and the ability to achieve erection during copulation. The inability to deposit semen plugs may derive from two possible sources: 1) perturbations in ejaculatory abilities that leave overall erectile functioning intact [19, 20], or 2) erectile dysfunction that decreases the ability to achieve or maintain an erection during copulation. Monitoring erectile function in the copulating mouse is challenging because of the speed with which males transition from the initiation of the mount to intromission, however, due to the high-speed nature of our recordings, we were able to detect that erectile function in copulating *Pea3* null male mice is dramatically reduced.

**Fig 3:**
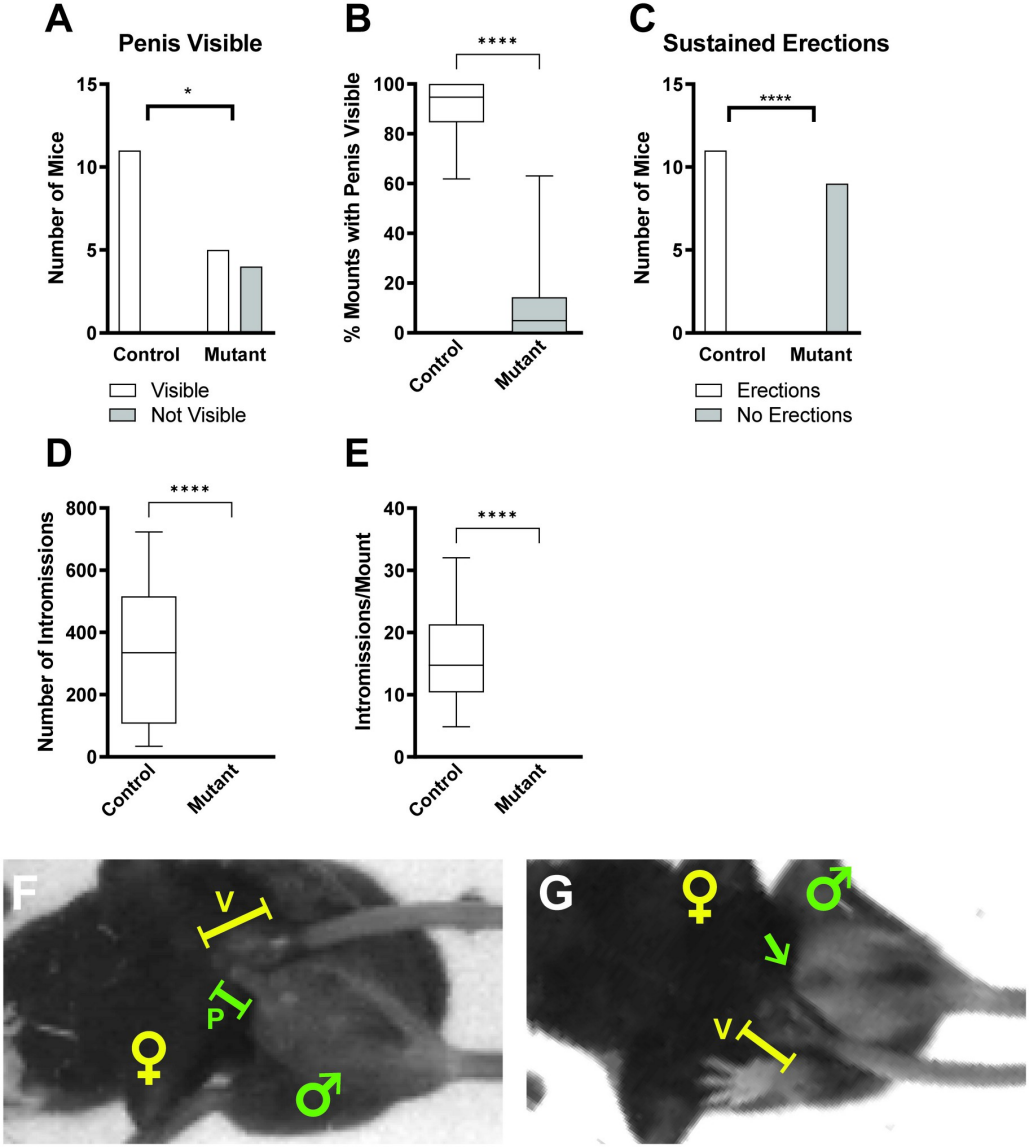
*Pea3* null males have greatly diminished erectile functioning during sexual behavior. (A) Of mounting mice, there are fewer *Pea3* null mice with the penis visible outside of the penile sheath compared to control mice (control: N = 11 mice; null: N = 9 mice; Fisher’s exact test, p < 0.026, *). (B) *Pea3* null mice exhibit a decreased percentage of mounts with the penis visible outside of the penile sheath during mounting behavior compared to control mice (control: 89.60±3.87, N = 11 mice; null: 11.83±6.72%, N = 9 mice; Mann-Whitney test, p < 0.0001, ****). (C) Of mounting mice, no *Pea3* null mice exhibit sustained erections during sex as opposed to all control mice doing so (control: N = 11 mice; null: N = 9 mice; Fisher’s exact test, p < 0.0001, ****). (D and E) *Pea3* null mice do not achieve intromission, as compared to control mice for the total number of intromissions performed (D; control: 331.20±68.32 intromissions, N = 11 mice; null: 0.0±0.0 intromissions, N = 9 mice; Mann-Whitney test, p < 0.0001, ****) and the number of intromissions per mount (E: control: 16.71±2.75 intromissions/mount, N = 11 mice; null: 0.0±0.0 intromissions/mount, N = 9 mice; Mann-Whitney test, p < 0.0001, ****). Representative images of the presence (control mice; F) or absence (*Pea3* null mice; G) of erectile function. Male and female positions are marked by green and yellow symbols, respectively. Green capped lines and arrows indicate the position of the penis (P), and yellow capped lines indicate the position of the vagina (V).

*Pea3* null males do not produce visible erections during the vast majority of mounting bouts (Fig 3A–3C and 3G; S1 Video). During the rare mounting bouts when the penis exits the penile sheath, it is only briefly (∼0.1 seconds) visible at end of trains of high frequency probing thrusts and is not properly targeted to the vagina to allow for intromission. In contrast, control males generate and maintain visible erections during the initiation of mounts and high frequency probing thrusts that immediately precede intromission (Fig 3A–3C and 3F; S2 Video). As such, *Pea3* null mice fail to achieve intromission, both when quantified as the overall number of intromissions and intromissions per mount (Fig 3D and 3E). Therefore, the erectile deficiencies present in *Pea3* null males during copulation offer the clearest explanation for their inability to deposit semen plugs in receptive females.

### Copulatory hip thrusting is perturbed in *Pea3* null mice

The abolishment of erectile functioning in *Pea3* null mice, despite otherwise largely intact gross sexual behaviors, offers an opportunity to address how erectile function and penile sensory feedback during copulation influences the number, timing, and variability of hip thrusts during mounting. Taking advantage of the high temporal resolution of our data recordings, we classified behaviors by inter-thrust timing, or the time it takes from the start of one hip thrust to the start of the next hip thrust, as probes, intromissions, or pauses (see Methods).

**Fig 4:**
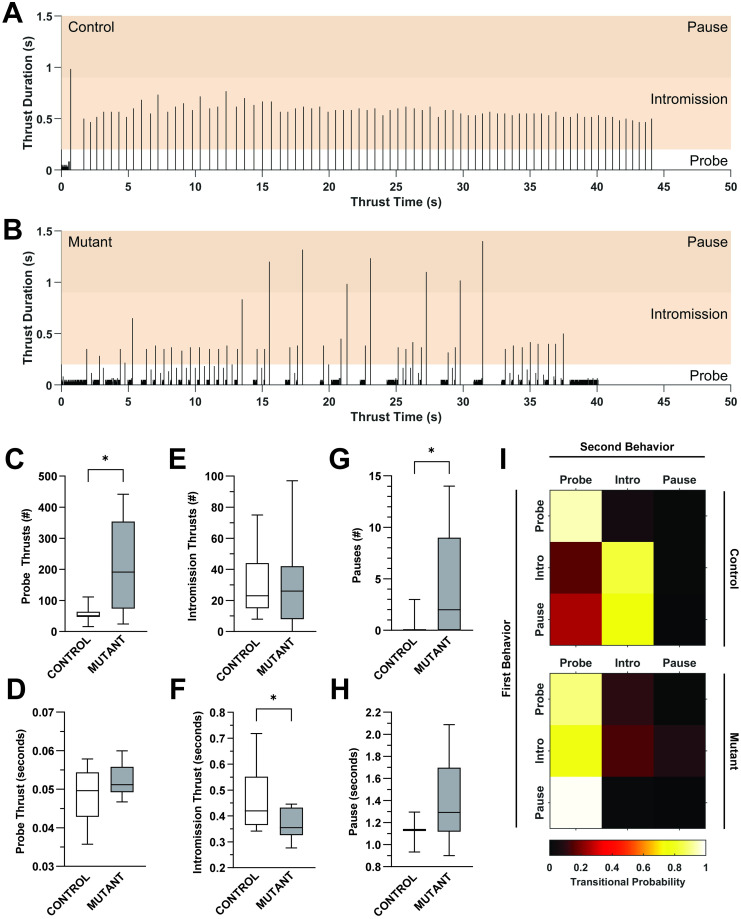
The organization and timing of thrusting behaviors is perturbed in *Pea3* null mice. (A and B) Representative thrusting patterns for a single mount for both a control (A) and *Pea3* null (C) mouse. Each line indicates a single thrust (duration and timing within the mounting bout). Color boxes indicate categorization of thrusts by thrust duration (probe: <0.2s; intromission: >0.2s and <0.9s; pause: >0.9s). (C and D) The number of probing thrusts per mount increases in *Pea3* null vs control mice (C; control: 57.36±7.884 thrusts, N = 11; null: 211.3±50.25 thrusts, N = 9; Welch’s t test, p < 0.0155, *), but the mean duration of probing thrusts is unchanged (D; control: 0.048±0.002 seconds, N = 11; null: 0.052±0.001 seconds, N = 9; Student’s t test, p < 0.1201, ns). (E and F) The number of intromission thrusts per mount does not change (E; control: 29.45±6.354 thrusts, N = 11; null: 29.44±9.881, N = 9; Mann-Whitney test, p < 0.8235, ns), however, the mean duration of intromission thrusts is shorter in *Pea3* null vs control mice (F: control: 0.460±0.034 seconds, N = 11; null: 0.369±0.020, N = 9; Mann-Whitney test, p < 0.0465, *). (G and H) The number of pauses increases in *Pea3* null vs control mice (G; control: 0.3636±0.2787; N = 11; null: 4.556±1.749, N = 9; Mann-Whitney test, p < 0.0199, *), but the mean duration of pauses is unchanged (H: control: 1.120±0.104 seconds; N = 3; null: 1.388±0.165 seconds, N = 6; Student’s t-test, p < 0.3221, ns). (I) Transitional probability diagram for control and *Pea3* null mice.

Compared to control mice, the thrusting pattern of *Pea3* null mice is clearly disrupted, with changes occurring in the number, duration, and sequential distribution of thrusting behaviors within a single mounting bout (Fig 4A and 4B). In *Pea3* null mice, there is an increase in the number, but not duration, of probing thrusts and pauses (Fig 4C, 4D, 4G and 4H). Conversely, *Pea3* null mice exhibit no change in the number of intromission-like thrusts, however, thrust duration is shorter than for control mice (Fig 4E and 4F). These data indicate that while the number and duration of specific thrusting behaviors within a mounting bout are changed, *Pea3* null mice are still capable of initiating the full range of thrusting behaviors.

Lastly, we observed that the temporal organization of the thrusting sequence in *Pea3* null males was highly disordered when compared to control males. In *Pea3* null males, probing thrusts and pauses were often interspersed between intromission-like thrusting patterns, whereas control males quickly transitioned into stable intromission thrusting patterns after an initial bout of probing thrusting (Fig 4A and 4B). We confirmed this observation by quantifying the transitional probability of specific thrusting behaviors (i.e., the probability that an intromission occurs when the preceding behavior was a probe, etc.). In control mice, we find that probing and intromission thrusts are tightly grouped in sequence, with a probe most likely being followed by a probe (94%; Fig 4I, top), and an intromission most likely being followed by another intromission (83%; Fig 4I, top). Strikingly, the sequential association of thrusting behaviors is broken in *Pea3* null mice, where for any type of thrusting behavior, a probe is the most likely subsequent thrusting behavior (89% for probe; 78% for intromission; 99% for pause; Fig 4I, bottom). These data indicate that the expression of *Pea3* contributes to controlling the transition between stable hip thrusting states, either indirectly through mediating erectile function or directly through involvement in organizing the motor control of hip thrusting patterns.

## Discussion

Here, we set out to assess *Pea3* null male mice for an array of potential deficiencies in male sexual behaviors that may occur during active copulation. We employed a sexual behavior arena that allows for the viewing of mounts, thrusts and intromissions from the mouse underside, granting a clear view of the penis during sexual activity [11]. We coupled this behavioral assay with high-speed, high-resolution video recording that allows for a detailed post-hoc analysis of sexual behaviors. We found that *Pea3* null male mice have greatly diminished erectile function during copulation, and therefore never achieve intromission, despite displaying sufficiently normal gross sexual motor behaviors (i.e., mounting, thrusting, and ejaculatory behavior).

How could loss of *Pea3* contribute to erectile dysfunction during copulation? For wildtype males, the pudendal muscles, the bulbocavernosus (BC) and ischiocavernosus (IC), contract synchronously during intromission, which increases intracavernosal pressure to achieve the erectile rigidity necessary for vaginal insertion [22]. During ejaculation, the activity of the BC and IC muscles shifts from synchronous to alternating, which turns the urethra into a pump and drives the expulsion of semen [22]. After excision of the IC muscle, successful intromissions are greatly reduced, however males continue to mount, thrust, and display ejaculatory patterns [21]. Excision of the BC muscle has little effect on erectile function, but reduces fertility during mating assays, which is suggested to be due to improper plug placement during ejaculation [16]. Similarly, transection of the motor branch of the pudendal nerve, which supplies motor efferent innervation for IC and BC muscles, results in a decrease in intromissions and ejaculations, a large increase of extravaginal intromission patterns, and an inability to gain erections during reflexive tests [23]. Firstly, the greatly diminished erectile function and extravaginal penile targeting during thrusting behaviors of *Pea3* null mice is similar to the phenotypes produced by the excision of the IC and BC muscles, or the transection of motor afferents that supply them. Secondly, the brief and poorly timed periods of visible erectile activity witnessed in *Pea3* null males suggest insufficient control over the timing of increases in intracavernosal pressure necessary for intromission, which is governed by the pudendal muscles and upstream sexual circuitry in the spinal cord [24]. *Pea3* is expressed within the brain, spinal cord, and dorsal root ganglia [8, 10, 12]. In conclusion, our data further support the theory of Laing *et al*. that expression of *Pea3* within the nervous system could play a role in organizing male sexual behavior [8].

The erectile deficiency witnessed in *Pea3* null male mice offers an opportunity to address the role of erectile function in organizing copulatory hip thrusting patterns. Our data show that, concurrent with the loss of erectile function, that there are dramatic alterations in hip thrusting patterns in *Pea3* null males. Control mice clearly show bi-stable patterns of thrusting frequencies (i.e., transitioning from a stable probing state to a stable intromission state). This bi-stable pattern is disrupted in *Pea3* null mice, which indicates that sensory feedback from intravaginal contact might contribute to the creation stable states of repetitive and uninterrupted intromission thrusts. Many studies have sought to identify the role of erectile sensory feedback on the organization and execution of mouse sexual motor behaviors, either by surgically inducing impotence [21, 23, 25], the application of potent topical local anesthetics to the penis [22, 25–27], or by limiting access to the vagina [26, 28]. Contrary to our results, however, in rats, sensory denervation of or the application of lidocaine to the penis does not block intromissions or disrupt hip thrusting patterns during mounts [25, 26], indicating that hip thrusting patterns during mounting can be generated sans sensory information from the penis. Therefore, a second and potentially more interesting conclusion is that the expression of the *Pea3* gene may be necessary for the proper function of neurons that modulate oscillatory hip thrusting patterns or control locomotor state transitions and stability during sexual behaviors [29].

There are few comparable studies within the literature that describe genetic mutations with similarly severely diminished erectile functioning during copulation, highlighting the novelty of the *Pea3* null male phenotype. The closest phenotypic match to *Pea3* null males are *TGF1beta* null male mice, which are infertile and do not deposit semen plugs during sexual behavior assays [30]. However, *TGF1beta* null males are able to achieve erections naturally (via self-grooming) despite clear deficits in the structural integrity of the penis [31], an issue which is not present in *Pea3* null males [8]. Additionally, there are many other genetic mutations in mice that lessen or abolish the deposition of semen plugs during short, monitored copulation assays, however, do not render males infertile in paired mating assays of longer duration (weeks to months) [7, 32–35], indicating the presence of sufficient erectile function to reproduce, again, in contrast to *Pea3* null males [8]. The transcription factor *Single-Minded Family BHLH Transcription Factor 1* (*SIM1*) has recently been linked to erectile dysfunction in humans [6], but in mouse models the direct knockout of the *SIM1* gene has yet to be assessed for erectile dysfunction [36]. *SIM1* is necessary for the development of hypothalamic neurons in the leptin/melanocortin pathway [36, 37]. Homozygous *SIM1* knockout mice die perinatally [38] and postnatal deletion of *SIM1* in mice leads to hyperphagic obesity [39], complicating the search for an erectile deficiency directly linked to altered *SIM1* expression. These studies highlight the novelty of the *Pea3* mutant phenotype, as homozygous *Pea3* null male mice are viable, display largely unaltered appetitive and consummatory male sexual behaviors, yet display greatly diminished erectile function during copulation. Interestingly, *Pea3*, also known as *ETS Variant Transcription Factor 4* (*Etv4*), has been linked to urological cancers in humans, however, has not been investigated as a cause for erectile dysfunction [40]. Therapeutics that target *Pea3*-linked cancers may offer novel pharmacological treatments for erectile dysfunction [41].

Future studies are necessary to interrogate the molecular mechanisms of *Pea3* in controlling erectile function during copulation. Such insights could potentially aid in the discovery of treatments for erectile dysfunction in patients [3].

## Supporting information

S1 Video *Pea3* null males have greatly diminished erectile functioning during sexual behavior. *Pea3* null males fail to generate erections during active copulation. Mounting and thrusting behavior is displayed at 1X and 1/10X video playback speed.
(MP4)

S2 Video Control males exhibit normal erectile functioning during sexual behavior. Control males generate erections during active copulation. Mounting and thrusting behavior is displayed at 1X and 1/10X video playback speed.
(MP4)
